# Concordance of HbA1c, creatinine, LDL cholesterol, HDL cholesterol, and total cholesterol between venous and self‐collected capillary blood samples sent by mail

**DOI:** 10.1111/jdi.70359

**Published:** 2026-06-16

**Authors:** Linda Hoffmeister, Christopher Schuchardt, Angelika Hafke, Eva Hummers, Alexandra Dopfer‐Jablonka, Georg M. N. Behrens, Andrea Stölting, Louis Kuhnke, Julie Schanz, Frank Müller, Christine Happle, Dominik Schröder

**Affiliations:** ^1^ Department of General Practice University Medical Center Göttingen Germany; ^2^ Department of Clinical Chemistry and Interdisciplinary UMG Laboratory University Medical Center Göttingen Göttingen Germany; ^3^ Department of Rheumatology and Immunology Hannover Medical School Hannover Germany; ^4^ German Center for Infection Research (DZIF), partner site Hannover‐Braunschweig Braunschweig Germany; ^5^ Interdisciplinary UMG‐Laboratories and Department of Hematology and Medical Oncology University Medical Center Göttingen Göttingen Germany; ^6^ Department of Family Medicine Michigan State University Grand Rapids Michigan USA; ^7^ Department of Pediatric Pneumology, Allergology, and Neonatology Hannover Medical School Hannover Germany; ^8^ German Center for Lung Research, DZL‐BREATH Hannover Germany

**Keywords:** Capillary blood, HbA1c, Self‐collection

## Abstract

**Introduction/Aims:**

Self‐collected capillary blood sampling with postal shipment may improve access to routine monitoring of HbA1c, creatinine, and lipid parameters, but evidence on concordance with venous samples under real‐world transport conditions remains limited. This study evaluated the concordance of HbA1c, creatinine, and lipid parameters between self‐collected mailed capillary samples, mailed venous samples, and courier‐transported venous samples.

**Material and Methods:**

In this cross‐sectional study, participants scheduled for a blood draw in a general practice office self‐collected a capillary blood sample using the Tasso+ device and provided additional venous blood samples. One venous sample was sent by mail (VMS) with the capillary blood sample (CMS). The other venous sample was couriered directly to the laboratory (VCS). HbA1c, creatinine, LDL, HDL, and total cholesterol were measured.

**Results:**

Samples of 105 participants were analyzed. High concordance was observed between CMS and VCS for HbA1c (absolute mean bias = 0.028%, relative mean bias = 0.463%, concordance correlation coefficient [CCC] = 0.999) and creatinine (absolute mean bias = 0.037 mg/dL, relative mean bias = 4.815%, CCC = 0.972). VMS showed high agreement with VCS for HbA1c (absolute mean bias = 0.028%, relative mean bias = 0.450%, CCC = 0.998) and creatinine (absolute mean bias = 0.026 mg/dL, relative mean bias = 3.320%, CCC = 0.990). Lipid parameters demonstrated good concordance for CMS and VMS compared to VCS, with relative bias values ranging from 3.0% to 5.7% and CCCs >0.95.

**Conclusions:**

Self‐collected mailed capillary blood samples provide comparable HbA1c, creatinine, and lipid parameters to venous samples, even when sent by standard mail. Capillary self‐sampling is a reliable method for remote monitoring of these parameters in diabetes management and screening.

## INTRODUCTION

Regular monitoring of HbA1c, creatinine, and lipid parameters is essential for both effective diabetes management and comprehensive cardiovascular risk assessment. HbA1c reflects average blood glucose levels over 2–3 months and is considered the primary metric for assessing glycemic control^1^. Creatinine is a marker of kidney function and is used to monitor the development and progression of diabetic nephropathy^2^, ^3^, while lipid parameters (LDL, HDL, and total cholesterol [total Chol]) are essential for cardiovascular risk assessment, particularly given that cardiovascular disease represents the leading cause of morbidity and mortality in this population^4^, ^5^. Current guidelines recommend HbA1c assessment at least twice a year (quarterly for diabetic patients not meeting targets or with recent treatment changes)^1^ and annual monitoring of creatinine and lipid parameters^6^.

Despite these monitoring recommendations, adherence to diabetes follow‐up appointments with blood testing remains challenging^7^, ^8^, ^9^. Multiple barriers contribute to the lack of in‐practice blood sampling, including limited clinic opening hours for working individuals^10^, difficulties accessing healthcare facilities due to frailty or immobility among older adults^11^, and restricted access to the healthcare system, particularly for patients with a migrant background^12^ or unclear insurance status^9^. Further barriers include limited availability of healthcare services for individuals living in rural areas with restricted access to care and for people with lower health literacy^13^. These access barriers contribute to insufficient glycemic control, delayed treatment adjustments, and increase the risk of diabetes‐related complications^8^, ^14^.

Self‐blood collection (SBC) systems enable at‐home sampling without the need for clinic visits^15^, ^16^. SBC approaches may improve adherence to monitoring guidelines and enable timelier treatment adjustment. Additionally, SBC is less painful compared to venous sampling, potentially reducing barriers to testing^17^, ^18^, ^19^. Recently introduced upper arm devices (e.g., Tasso+, TAPII) can collect large blood volumes (up to 0.5 mL)^20^, ^21^, allowing completion of all recommended diabetic check‐up bloodwork from a single capillary sample. However, the concordance of capillary samples with venous samples for multi‐parameter assessment requires evaluation, particularly when shipped by standard postal service.

Previous studies have demonstrated high concordance between self‐collected capillary and venous HbA1c measurements^22^, ^23^, ^24^ including those shipped by regular mail to the laboratory^25^. For creatinine, available evidence is limited. Previous studies have reported differing levels of agreement between capillary and venous measurements, which may be attributable to differences in analytical methodology (Jaffe versus enzymatic assays)^26^, ^27^. Stability of lipid parameters in mailed capillary samples using standard postal service remains scarce. Moreover, most prior studies involved controlled laboratory settings or immediate sample processing rather than real‐world home‐based collection and standard postal shipping.

The aim of the study is therefore to investigate the concordance of HbA1c, creatinine, and lipid parameters between self‐collected capillary samples and venous samples in a general practice setting. Participants performed SBC independently and shipped samples to the laboratory by standard mail, reflecting realistic implementation conditions. To distinguish the effects of the collection methods from those of postal transport, we compared both mailed capillary and mailed venous samples against venous samples transported directly to the laboratory by courier.

## MATERIALS AND METHODS

### Study design

The “Blood‐it‐yourself Study” is a cross‐sectional observational study, examining the feasibility of a capillary self‐blood collection system using the Tasso+® device (Tasso Inc., Seattle, WA, USA). The study was registered in the German Clinical Trials Register (DRKS00033172) and received approval through the research ethics board of the University Medical Center Göttingen (23/12/23). Participant recruitment took place in two general practices located in a rural area of Central Germany. A separate paper on SBC‐associated pain and usability has been recently published^28^.

### Inclusion and exclusion criteria

Inclusion criteria were (a) participant age 18 years or older; (b) indication for a blood draw; (c) proficiency in German. Exclusion criteria were (a) inability to consent; (b) a contraindication for capillary blood collection (e.g., extensive scarring or skin infection on drawing site); and (c) the presence of a medical emergency requiring immediate action. Individuals with and without diagnosed diabetes were eligible to participate as self‐collection systems may be used for diabetes screening and monitoring of prediabetes in addition to established diabetes management.

### Study flow and data collection

**Figure 1:**
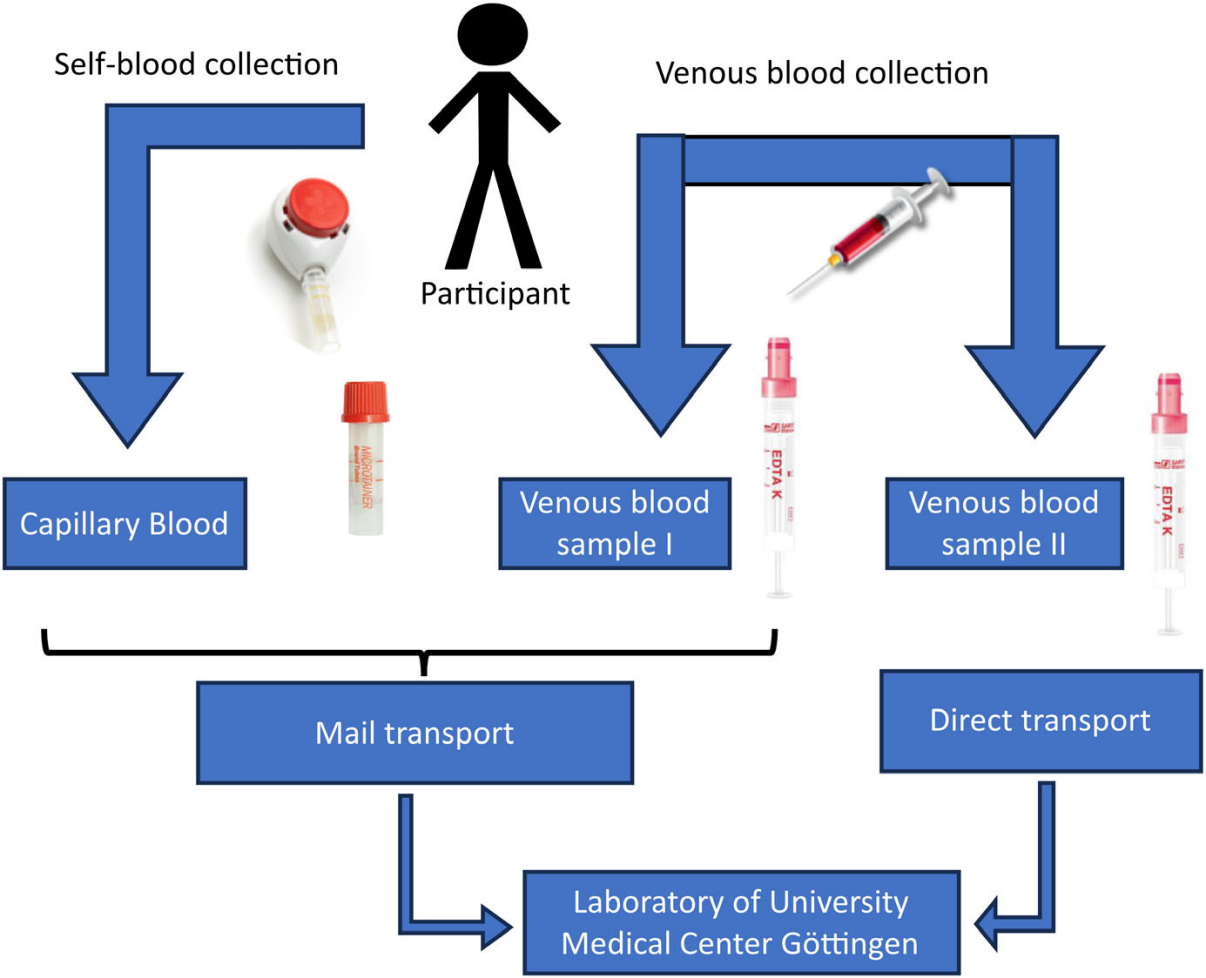
Study conduction inclusive sample logistics. CMS, capillary mail sample; VMS, venous mail sample; VCS, venous courier sample.

After obtaining informed consent, a capillary blood sample up to 0.5 mL (BD Microtainer® Lithium heparin [Becton, Dickinson and Company, NJ, USA]) and two 7.5 mL venous blood samples (S‐Monovette® Lithium Heparin [SARSTEDT AG &Co. KG, Nürmbrecht, Germany]) were collected within a 10‐min timeframe. SBC was performed using the Tasso+® device. Instructions and all other equipment needed (heat pad, band‐aid, etc.) were provided to the participants. Participants who were unable to perform the SBC independently received assistance from study personnel. After the procedure, participants were asked to complete a questionnaire on sociodemographics, health status, and self‐blood collection experience. Finally, a package with the capillary blood sample (CMS = capillary mail sample) and venous blood sample (VMS = venous mail sample) was prepared by study personnel, which participants could either drop into a mailbox or submit at a post office. The prepaid package was addressed to the study laboratory at UMG. One venous blood sample (VCS = venous courier sample) was transported directly to the UMG laboratory by courier on the day of sample collection (transportation time <4 h) and analyzed immediately upon arrival (Figure 1).

Packages sent by mail were unpacked, processed, and measured within 2–3 h upon arrival at the laboratory on working days. If participants' packages arrived over the weekend, they were stored uncentrifuged at 4°C until the next working day.

### Measures

Participant characteristics including age, sex, body mass index, and comorbidities were assessed using a pen/paper questionnaire. Health‐related quality of life was measured using the German version of the EQ‐5D‐5L instrument^29^ and the respective value set to calculate index scores^30^. The questionnaire also captured information on the feasibility of self‐blood collection, including whether participants required assistance from study personnel.

### Laboratory methods

HbA1c was measured via high‐performance liquid chromatography (HPLC) using a Tosoh automated glycohemoglobin analyzer HLC‐723G8 (Tosoh K.K., Tokyo, Japan) in standard analysis mode. To prevent contamination of the HPLC column, Li‐heparin whole blood was diluted 1:200 using Tosoh HbA1c dilution buffer prior to analysis. The proportion of HbA1c relative to total hemoglobin was then determined. For analyses of creatinine and lipid parameters, plasma was obtained by centrifugation (15 min, 2,500 ***g***, room temperature). Measurements were performed using Abbott's Creatinine, Ultra HDL, Direct LDL and Cholesterol enzymatic assays on an Architect c16000 analyzer (Abbott Laboratories, Abbott Park, IL, USA).

### Sample size

No formal sample size calculation was performed for this exploratory study. At the time of study planning, there was limited evidence on sample quality and analyzability after standard postal transport. In addition, there was uncertainty regarding the success rate of self‐collection using the Tasso+ device, the volume of capillary blood obtained per participant under real‐world primary care conditions, and the expected loss‐to‐follow‐up rate in a primary care setting. Previously published validation studies reported highly heterogeneous correlation coefficients between capillary and venous measurements, precluding reliable a priori power calculation^31^. Therefore, a target sample size of approximately 100 participants was determined based on feasibility considerations and consistency with prior studies and was considered sufficient to detect even low correlation coefficients while accounting for potential sample loss and non‐analyzable specimens.

### Measures to reduce bias

The within‐subject design, in which each participant provided all three blood samples (CMS, VMS, VCS) at the same time point, ensured that each participant served as their own control. This inherently controlled for between‐subject variability and minimized bias due to individual differences such as age, sex, comorbidities, medications, and physiological state, thereby reducing the need for statistical adjustment for confounding variables.

### Statistical analysis

Participants' characteristics were presented as absolute number of participants with its corresponding proportion for categorical variables or median and 25th and 75th percentile for numeric variables. Concordance between two blood sampling methods was investigated using the absolute and relative mean bias, accuracy of the parameters based on the threshold values (HbA1c [%]: <5.7, 5.7–6.4, >6.4^32^; creatinine [mg/dl] males <0.7, 0.7–1.2, >1.2 and females <0.6, 0.6–1.1, >1.1^33^; LDL [mg/dl]: <100, 100–129; >129; HDL [mg/dl]: <40; 40–60; >60; total Chol [mg/dl]: <200; 200–239; >239), correlation coefficients (Lin's concordance correlation coefficient (CCC), Spearman)^34^, ^35^, and regression analyses (Deming regression and Passing‐Bablok regression^36^). In addition, the relative mean bias for each participant was categorized as to whether it met the requirements of the German Medical Association's guideline for quality assurance of laboratory medical examinations (tolerable relative deviation for HbA1c, creatinine, LDL, HDL and total Chol is 3.0%, 11.5%, 9.0%, 13.0% and 7.0%, respectively)^37^. CMS and VMS were compared with the gold standard of the VCS. In addition, the CMS was also compared with the VMS to examine whether there is a possible difference due to the use of capillary blood compared to venous blood. The number of cases analyzed differs between the blood collection and shipping methods and also the analyzed laboratory parameters, as in some subjects, not enough capillary blood volume could be collected with the Tasso+ to determine all laboratory values.

The results are presented using scatterplots with the Deming regression line and Bald‐Altmann plots. All statistical analyses and illustrations were performed using R (version 4.4.0) with the packages *mcr* ^38^, *caret* ^39^ and *ggplot2* ^40^.

## RESULTS

**Figure 2:**
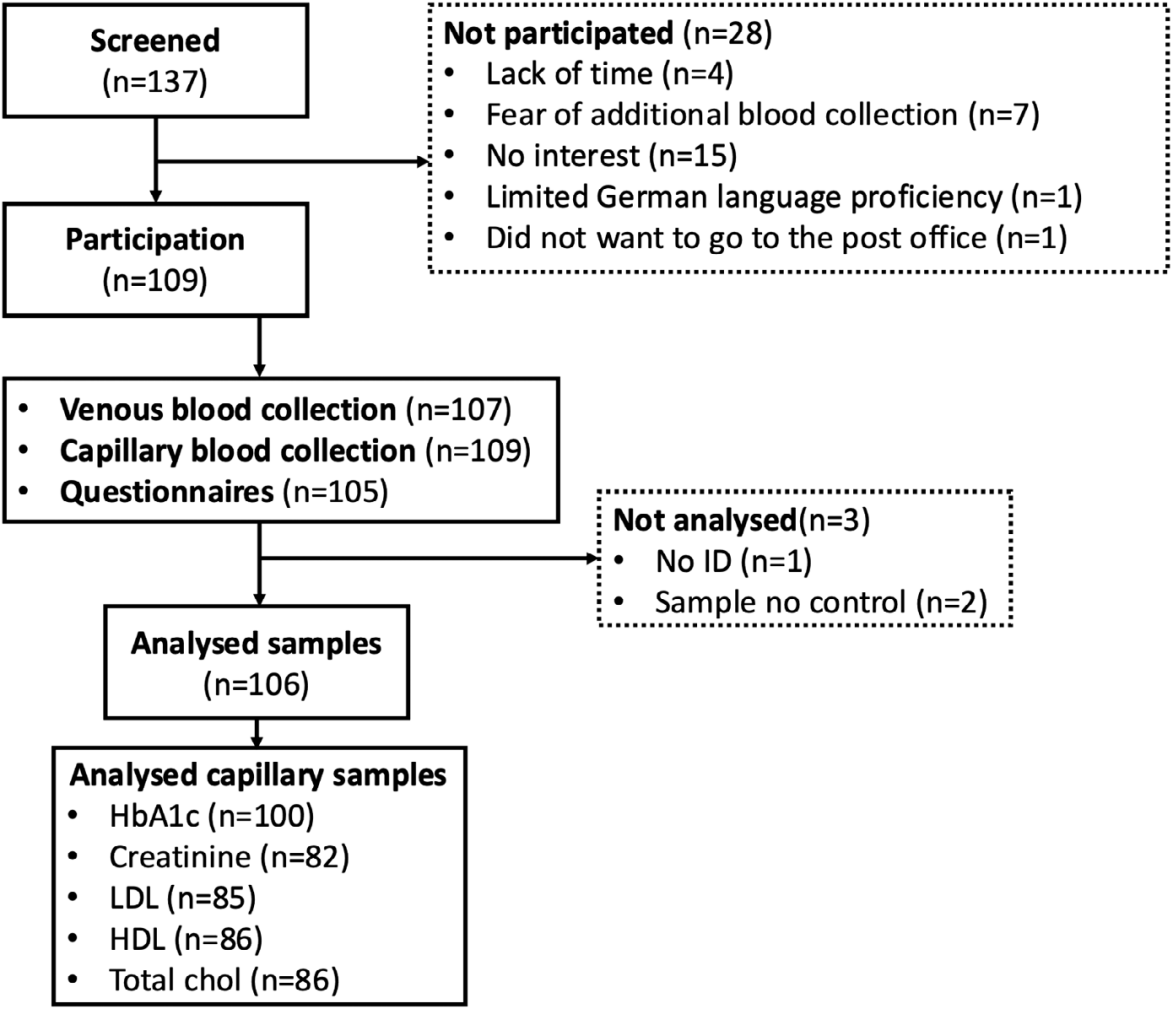
Flow chart of participant inclusion.

In total, 137 potential participants were approached, of whom 108 were enrolled in our study. In two participants, no venous blood sample was drawn, and in one participant, the IDs could not be matched and were therefore excluded from analyses. From the remaining 106 participants, blood volume from CMS was sufficient in 100, 82, 85, 86, and 86 participants to determine HbA1c, creatinine, LDL, HDL, and total Chol, respectively (Figure 2).

### Sample description

Participant characteristics are presented in Table 1.

**Table 1:** Participant characteristics

Age (missing *n* = 4)	Median (Q25 – Q75)	57.0 (41.0–65.0)
Sex (missing *n* = 10)	Female (%)	58 (55.2)
BMI (kg/m^2^) (missing *n* = 4)	Median (Q25 – Q75)	26.3 (22.8–30.3)
	<18.5 (%)	4 (4.0)
	18.5–25.0 (%)	36 (35.6)
	>25.0 (%)	61 (60.4)
Comorbidities (missing *n* = 3)	Diabetes (%)	17 (16.7)
	Lymphoedema (%)	2 (2.0)
	Cancer (%)	5 (4.9)
	Autoimmune or rheumatic condition (%)	11 (10.8)
	Coronary heart disease or heart failure (%)	15 (14.7)
	Psychiatric condition (%)	5 (4.9)
EQ‐5D‐5L index^1^ (missing *n* = 9)	No impairments (%)	30 (31.3)
	Median (Q25 – Q75)^1^	0.91 (0.83–1.00)
HbA1c (%)*	Median (Q25 – Q75)	5.7 (5.4–6.1)
	<5.7 (%)	47 (44.8)
	5.7–6.4 (%)	41 (39.0)
	>6.5 (%)	17 (16.2)

Scale 0–1, 0 = worst health, 1 = best health. BMI, body mass index.

* HbA1c determined from venous courier sample (VCS).

Mailed samples (CMS and VMS) arrived at the laboratory after a mean time of 40.2 h (SD = 30.1 h); eight samples exceeded 120 h transport time. No samples showed hemolysis above the analytical threshold for creatinine (1,000 mg/dL), LDL (500 mg/dL), HDL (2,000 mg/dL), or total Chol (750 mg/dL).

### Concordance of HbA1c

**Figure 3:**
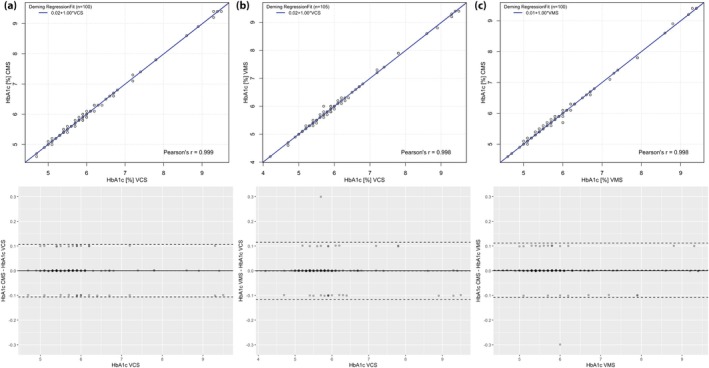
HbA1c – Scatter plot with Deming regression line (95% CI) with corresponding Bland–Altman plot. (a) CMS vs. VCS; (b) VMS vs. VCS; (c) CMS vs. VMS. CMS, capillary mail sample; VMS, venous mail sample; VCS, venous courier sample; dashed lines: two standard deviations from mean.

HbA1c showed high concordance between all sampling and shipping methods (Table 2). Maximum absolute deviation between methods was 0.1% (CMS vs. VCS), and 0.3% (VMS vs. VCS; CMS vs. VMS), with most participants showing deviations of ≤0.1% (Figure 3).

**Table 2:** Concordance measure of HbA1c between different blood collection and shipping methods

	CMS vs. VCS (*n* = 100)	VMS vs. VCS (*n* = 105)	CMS vs. VMS (*n* = 100)
Mean Bias			
Absolute^†^	0.028 (0.045)	0.028 (0.051)	0.024 (0.049)
Relative (%)^†^	0.463% (0.759)	0.450% (0.854)	0.396% (0.838)
Correlation coefficient			
Pearson^‡^	0.999 (0.998–0.999)	0.998 (0.998–0.999)	0.998 (0.998–0.999)
CCC^‡^	0.999 (0.998–0.999)	0.998 (0.998–0.999)	0.998 (0.998–0.999)
Regression			
Deming	Y = 0.025 + 0.996*VCS	Y = 0.021 + 0.996*VCS	Y = 0.012 + 0.998*VMS
Passing‐Bablok	Y = 0.000 + 1.000*VCS	Y = 0.000 + 1.000*VCS	Y = 0.000 + 1.000*VMS
Binary classification			
Accuracy^‡^ ^,^ ^§^	0.990 (0.946–1.000)	0.981 (0.933–0.998)	0.980 (0.930–0.998)
Proportion of cases inside tolerable relative deviation^‡^ ^,^ ^¶^	1.0 (1.0–1.0)	0.990 (0.948–1.0)	0.990 (0.946–1.0)

^†^ Mean (SD).

^‡^ With corresponding.

^§^ Categorized based on the cutoffs <5.7, 5.7–6.4, >6.4.

^¶^ Tolerable relative deviation is 3% for HbA1c (German Medical Association's guideline for quality assurance of laboratory medical examinations^37^).

### Concordance of creatinine

**Figure 4:**
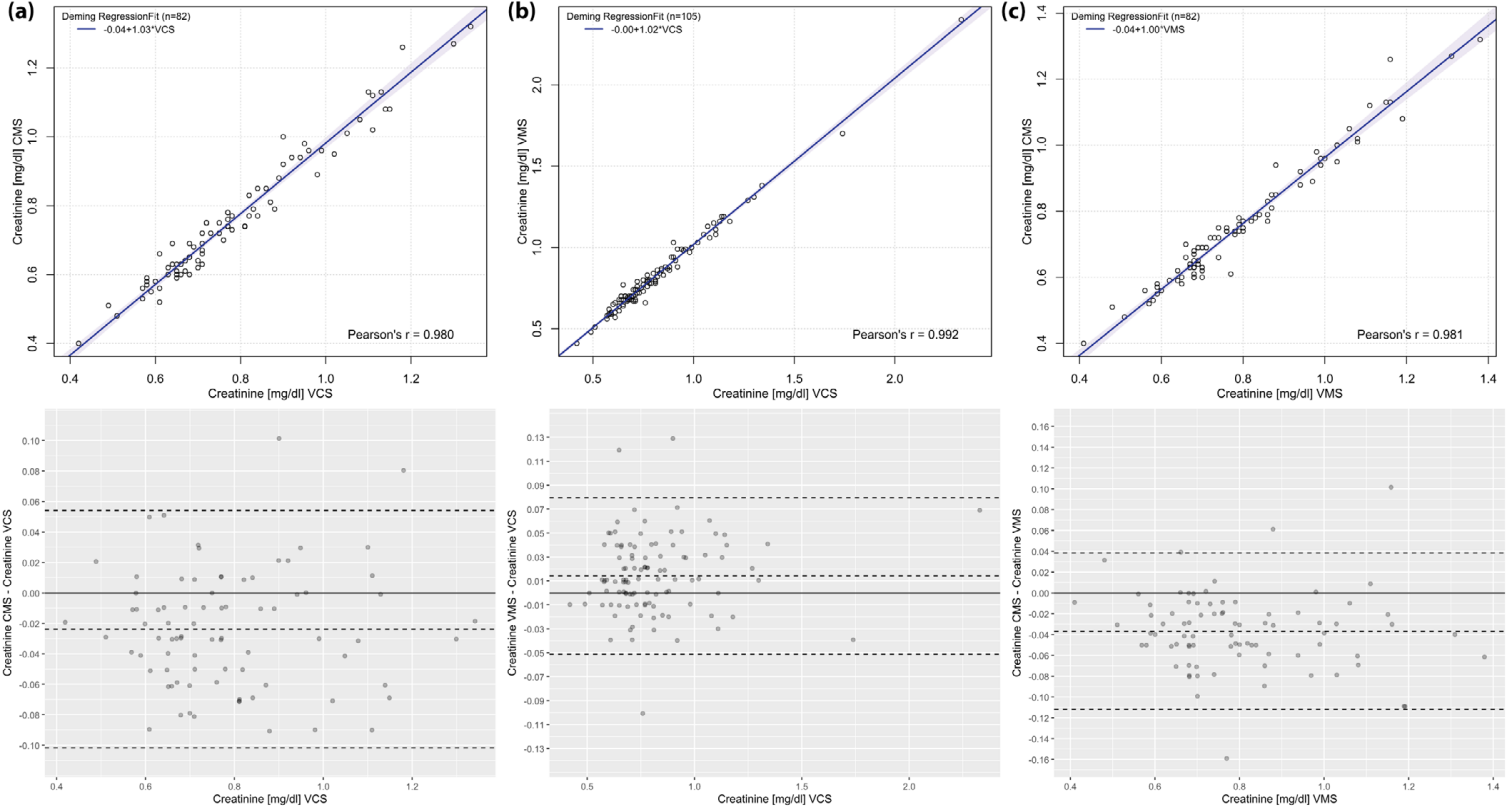
Creatinine – Scatter plot with Deming regression line (95% CI) with corresponding Bland–Altman plot. (a) CMS vs. VCS; (b) VMS vs. VCS; (c) CMS vs. VMS. CMS, capillary mail sample; VMS, venous mail sample; VCS, venous courier sample; dashed lines: two standard deviations from mean.

A high level of agreement was observed for creatinine between all compared blood collection methods and shipping methods. The highest agreement was observed between the two venous blood samples. The capillary blood samples showed a slightly lower agreement with the venous blood samples (Figure 4, Table 3).

**Table 3:** Concordance measure of creatinine between different blood collection and shipping methods

	CMS vs. VCS (*n* = 82)	VMS vs. VCS (*n* = 105)	CMS vs. VMS (*n* = 82)
Mean Bias			
Absolute (mg/dL)^†^	0.037 (0.027)	0.026 (0.024)	0.043 (0.030)
Relative (%)^†^	4.815 (3.391)	3.320 (3.123)	4.785 (3.444)
Correlation coefficient			
Pearson^‡^	0.980 (0.969–0.987)	0.992 (0.988–0.995)	0.981 (0.971–0.988)
Concordance correlation coefficient^‡^	0.972 (0.957–0.981)	0.990 (0.986–0.993)	0.964 (0.946–0.976)
Regression			
Deming	Y = −0.045 + 1.027*VCS	Y = −0.004 + 1.023*VCS	Y = −0.035 + 0.998*VMS
Passing‐Bablok	Y = −0.030 + 1.000*VCS	Y = −0.004 + 1.021*VCS	Y = −0.040 + 1.000*VMS
Binary classification			
Accuracy^‡^ ^,^ ^§^	0.935 (0.855–0.979)	0.959 (0.898–0.989)	0.934 (0.853–0.978)
Proportion of cases inside tolerable relative deviation^‡^ ^,^ ^¶^	0.976 (0.915–0.997)	0.971 (0.919–0.994)	0.951 (0.880–0.987)

^†^ Mean (SD).

^‡^ With corresponding 95% CI.

^§^ Categorized based on the cutoffs for males <0.7, 0.7–1.2, >1.2 and females <0.6, 0.6–1.1, >1.1.

^¶^ Tolerable relative deviation is 11.5% for creatinine (German Medical Association's guideline for quality assurance of laboratory medical examinations^37^).

### Concordance of LDL

**Figure 5:**
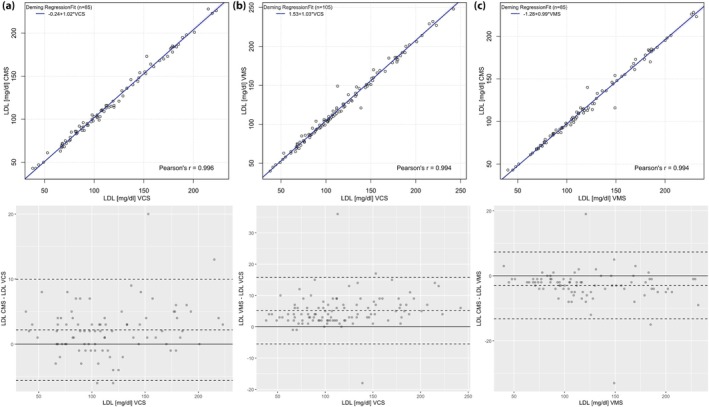
LDL cholesterol – Scatter plot with Deming regression line (95% CI) with corresponding Bland–Altman plot. (a) CMS vs. VCS; (b) VMS vs. VCS; (c) CMS vs. VMS. CMS, capillary mail sample; VMS, venous mail sample; VCS, venous courier sample; dashed lines: two standard deviations from mean.

LDL values showed a very high concordance between capillary and venous blood samples, with absolute mean biases below 6 mg/dL across all comparisons (Table 4, Figure 5). Correlation coefficients and CCC were consistently >0.98, indicating excellent agreement.

**Table 4:** Concordance measure of LDL between different blood collection and shipping methods

	CMS vs. VCS (*n* = 85)	VMS vs. VCS (*n* = 105)	CMS vs. VMS (*n* = 85)
Mean Bias			
Absolute^†^	3.200 (3.105)	5.524 (4.919)	3.847 (4.505)
Relative (%)^†^	3.045 (3.022)	5.041 (4.478)	3.248 (3.238)
Correlation coefficient			
Pearson^‡^	0.996 (0.995–0.998)	0.994 (0.991–0.996)	0.994 (0.990–0.996)
Concordance correlation coefficient^‡^	0.995 (0.992–0.997)	0.987 (0.982–0.991)	0.992 (0.987–0.994)
Regression			
Deming	Y = −0.244 + 1.021*VCS	Y = 1.527 + 1.031*VCS	Y = −1.279 + 0.986*VCS
Passing‐Bablok	Y = 1.009 + 1.009*VCS	Y = 1.425 + 1.025*VCS	Y = −0.928 + 0.986*VCS
Binary classification			
Accuracy^‡^ ^,^ ^§^	0.953 (0.884–0.987)	0.914 (0.844–0.960)	0.929 (0.853–0.974)
Proportion of cases inside tolerable relative deviation^‡^ ^,^ ^¶^	0.953 (0.884–0.987)	0.876 (0.794–0.930)	0.965 (0.893–0.991)

^†^ Mean (SD).

^‡^ With corresponding 95% CI.

^§^ Categorized based on the cutoffs <100, 100–129, >129.

^¶^ Tolerable relative deviation is 9% for LDL (German Medical Association's guideline for quality assurance of laboratory medical examinations^37^).

### Concordance of HDL

**Figure 6:**
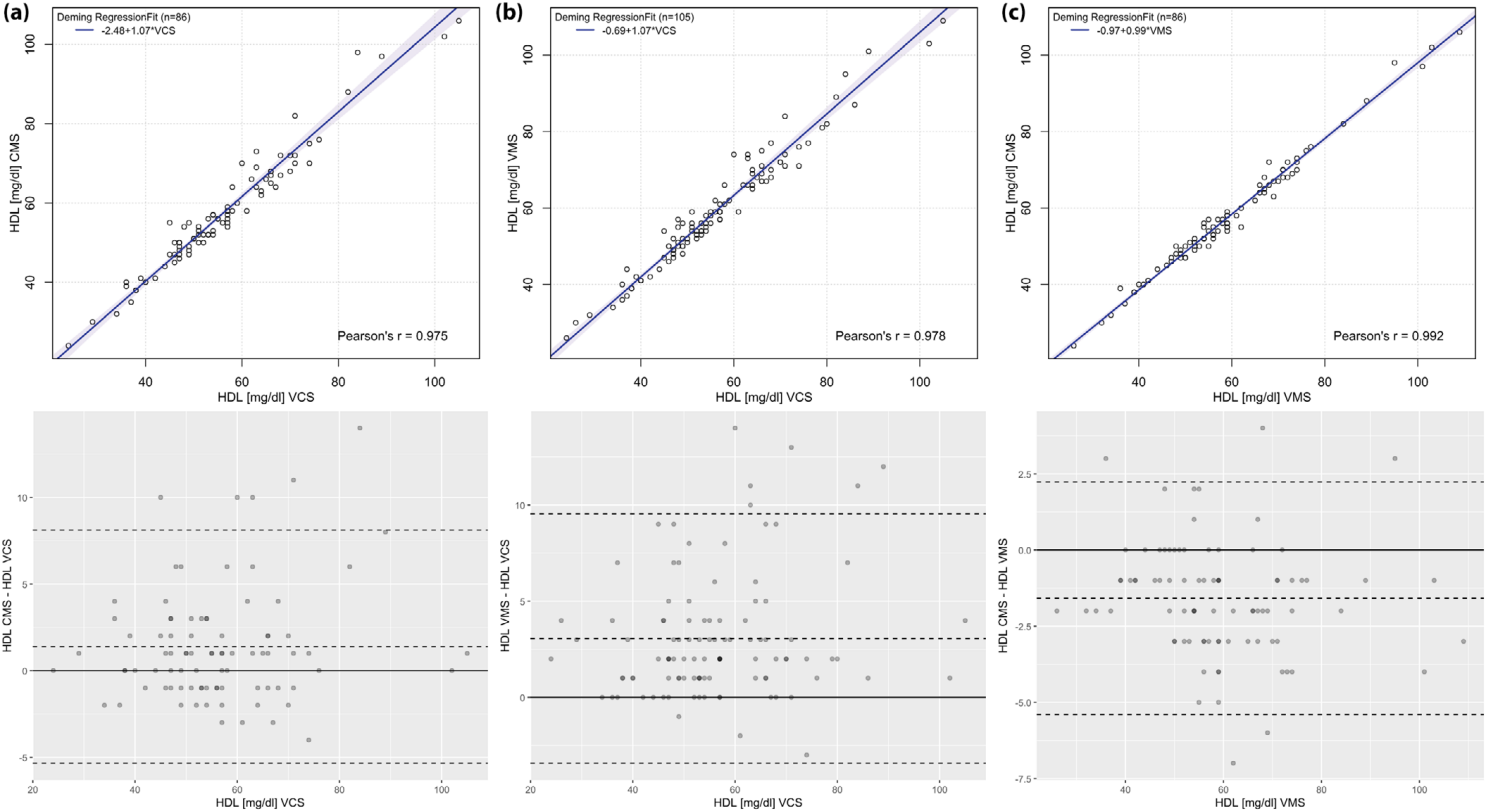
HDL cholesterol – Scatter plot with Deming regression line (95% CI) with corresponding Bland–Altman plot. (a) CMS vs. VCS; (b) VMS vs. VCS; (c) CMS vs. VMS. CMS, capillary mail sample; VMS, venous mail sample; VCS, venous courier sample; dashed lines: two standard deviations from mean.

For HDL, high concordance was observed between the different sampling and shipping methods (Table 5, Figure 6). Mean bias values were small, and correlation coefficients as well as CCCs exceeded 0.95 in all comparisons.

**Table 5:** Concordance measure of HDL between different blood collection and shipping methods

	CMS vs. VCS (*n* = 86)	VMS vs. VCS (*n* = 105)	CMS vs. VMS (*n* = 86)
Mean Bias			
Absolute^†^	2.407 (2.720)	3.171 (3.130)	2.000 (1.455)
Relative (%)^†^	4.242 (4.415)	5.707 (5.384)	3.448 (2.480)
Correlation coefficient			
Pearson^‡^	0.975 (0.962–0.984)	0.978 (0.967–0.985)	0.992 (0.988–0.995)
Concordance correlation coefficient^‡^	0.969 (0.954–0.979)	0.955 (0.937–0.968)	0.987 (0.980–0.991)
Regression			
Deming	Y = −2.482 + 1.069*VCS	Y = −0.694 + 1.067*VCS	Y = −0.965 + 0.990*VCS
Passing‐Bablok	Y = 1.000 + 1.000*VCS	Y = −0.020 + 1.039*VCS	Y = −2.000 + 1.000*VCS
Binary classification			
Accuracy^‡^ ^,^ ^§^	0.942 (0.870–0.981)	0.905 (0.832–0.953)	0.965 (0.901–0.993)
Proportion of cases inside tolerable relative deviation^‡^ ^,^ ^¶^	0.941 (0.863–0.978)	0.848 (0.761–0.908)	1.000 (0.947–1.000)

^†^ Mean (SD).

^‡^ With corresponding 95% CI.

^§^ Categorized based on the cutoffs <40, 40–60, >60.

^¶^ Tolerable relative deviation is 13% for HDL (German Medical Association's guideline for quality assurance of laboratory medical examinations^37^).

### Concordance of total cholesterol

**Figure 7:**
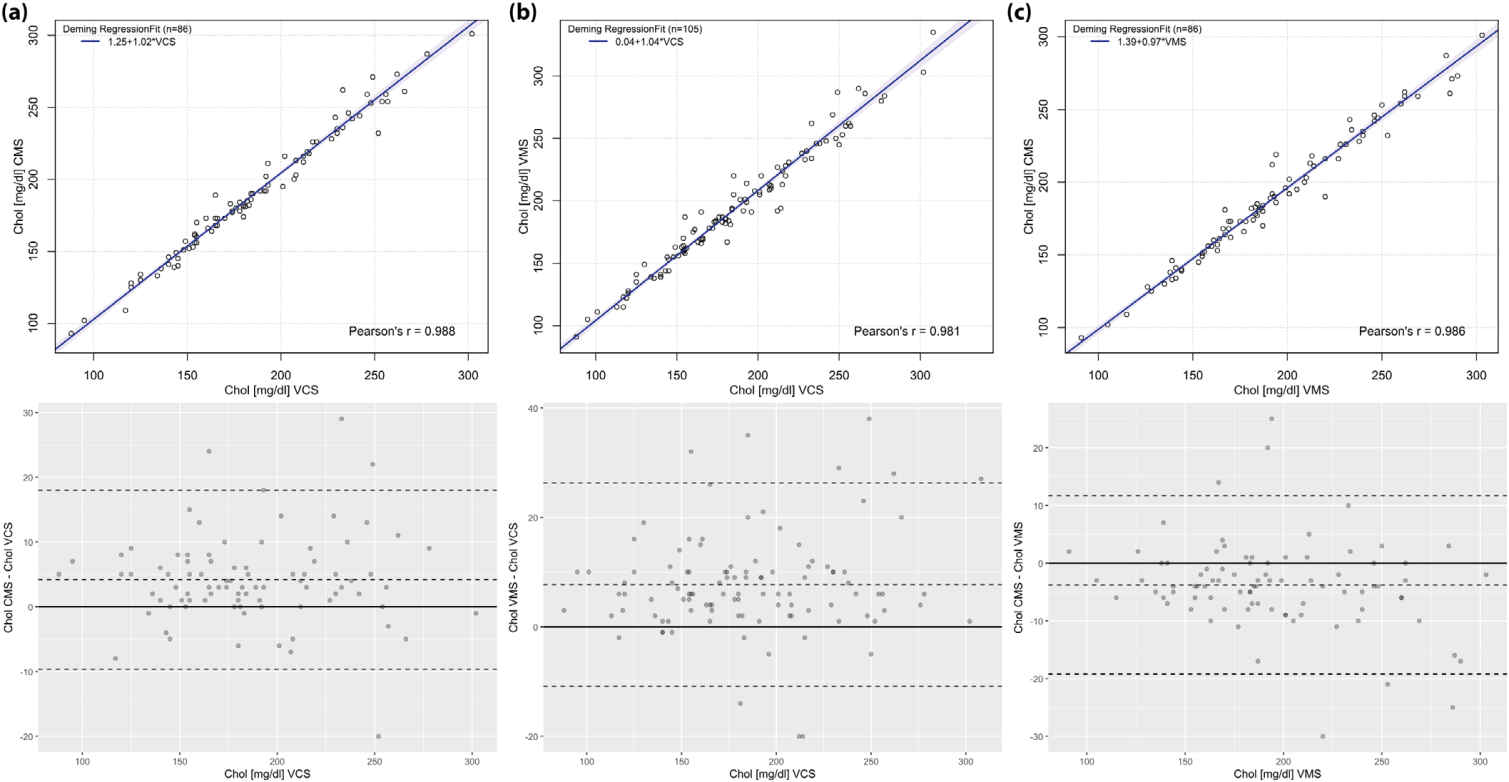
Total cholesterol – Scatter plot with Deming regression line (95% CI) with corresponding Bland–Altman plot. (a) CMS vs. VCS; (b) VMS vs. VCS; (c) CMS vs. VMS. CMS, capillary mail sample; VMS, venous mail sample; VCS, venous courier sample; dashed lines: two standard deviations from mean.

Total Chol measurements also demonstrated high levels of agreement between capillary and venous samples (Table 6, Figure 7). Absolute mean biases remained below 10 mg/dL, and concordance correlation coefficients indicated strong comparability across drawing and shipment methods.

**Table 6:** Concordance measure of total cholesterol between different blood collection and shipping methods

	CMS vs. VCS (*n* = 86)	VMS vs. VCS (*n* = 105)	CMS vs. VMS (*n* = 86)
Mean Bias			
Absolute^†^	5.837 (5.558)	9.124 (7.909)	6.221 (5.910)
Relative (%)^†^	3.219 (2.887)	5.023 (4.154)	3.177 (2.716)
Correlation coefficient			
Pearson^‡^	0.988 (0.981–0.992)	0.981 (0.973–0.987)	0.986 (0.978–0.991)
Concordance correlation coefficient^‡^	0.983 (0.975–0.989)	0.967 (0.954–0.977)	0.982 (0.973–0.988)
Regression			
Deming	Y = 1.245 + 1.016* VCS	Y = 0.036 + 1.041* VCS	Y = 1.391 + 0.974* VCS
Passing‐Bablok	Y = 3.000 + 1.000* VCS	Y = 3.137 + 1.018* VCS	Y = −1.637 + 0.987* VCS
Binary classification			
Accuracy^‡^ ^,^ ^§^	0.907 (0.825–0.959)	0.867 (0.786–0.925)	0.884 (0.797–0.943)
Proportion of cases inside tolerable relative deviation^‡^ ^,^ ^¶^	0.895 (0.806–0.948)	0.752 (0.657–0.829)	0.919 (0.834–0.964)

^†^ Mean (SD).

^‡^ Including 95% CI.

^§^ Categorized based on the cutoffs <200, 200–239, >239.

^¶^ Tolerable relative deviation is 7% for total cholesterol (German Medical Association's guideline for quality assurance of laboratory medical examinations^37^).

## DISCUSSION

Monitoring HbA1c, creatinine, and lipid parameters is essential in diabetes management, yet requires patients to visit healthcare facilities for venous blood draws. Capillary self‐blood collection sent by mail could address this barrier, but its validity under real‐world postal transport conditions has been insufficiently studied. In this study of 105 general practice patients, we demonstrate that self‐collected mailed capillary samples provide highly concordant results for all three parameter groups compared to venous samples, even after an average of 40 h in standard postal transport. By systematically comparing mailed capillary samples, mailed venous samples, and courier‐transported venous samples, we isolate the effects of sampling method and transport conditions, demonstrating that both are acceptable for clinical use.

These findings confirm and extend previous evidence on the comparability of capillary and venous blood for HbA1c testing^22^, ^24^, ^25^. For creatinine, previous capillary‐venous comparisons showed inconsistent results^26^, ^27^, likely due to differences in analytical methods (Jaffe vs. enzymatic assays). Our results using an enzymatic method demonstrate high concordance even after postal transport. For lipid parameters, this is the first evaluation of mailed capillary samples, showing good concordance despite greater variability than HbA1c.

An important finding of this study is the high concordance between mailed and courier‐transported venous samples (VMS vs. VCS). The relative bias for HbA1c was only 0.450% and for creatinine 3.320%, while lipid parameters showed relative bias values of 5.041% for LDL, 5.707% for HDL, and 5.023% for total Chol (tolerable deviations defined by the German Medical Association's guidelines for quality assurance of laboratory medical examinations: HbA1c 3.0%, creatinine 11.5%, LDL 9.0%, HDL 13.0%, total Chol 7.0%^37^). Mailed capillary samples showed slightly higher deviations compared to the venous courier sample (CMS vs. VCS), with relative bias of 0.463% for HbA1c, 4.815% for creatinine, and 3.0–4.2% for lipid parameters. Notably, all capillary HbA1c measurements remained within the tolerable deviation limits and 99% of capillary samples were correctly classified into the respective diagnostic categories (<5.7%, 5.7–6.4%, >6.4%). When comparing capillary and venous samples both sent by mail (CMS vs. VMS), comparable deviations were observed, suggesting that the observed differences can be attributed primarily to the capillary sampling technique rather than to the transport conditions. The absence of relevant hemolysis in all mailed samples despite an average transport time of 40.2 h (eight samples had transport times >120 h) indicates that standard postal transport may be sufficient for sample transport enabling use of SBC for diabetes monitoring. However, these findings are based on the German postal system, where regulations require that 95% of standard^41^.

Mailed capillary self‐sampling could improve adherence to diabetes care guidelines by eliminating practice visits solely for blood draws and enabling frequent and convenient testing^42^, ^43^, ^44^. Furthermore, SBC could not only support existing Disease Management Programs (DMP) for diabetes, but also allow them to be conducted through virtual visits^45^, ^46^. In addition, SBC is less invasive and painful compared to venous blood sampling^17^, ^31^, ^47^ which could reduce further barriers to regular monitoring. Although similar testing may also be feasible using fingerprick‐based collection methods, blood volume and pain levels vary substantially across collection technologies, with some lancet systems yielding lower sample volumes and higher pain scores than upper arm devices^20^, ^21^, ^48^. Beyond monitoring, SBC can also be used to facilitate diabetes screening, particularly relevant given that 22.8% of adults with diabetes in the United States remain untreated and undiagnosed^49^, with higher rates among minority populations^50^.

Before the implementation of SBC in clinical practice, several aspects need further evaluation. First, patients' and providers' acceptability should be assessed, including ease of use of the equipment, the clarity of instructions, and the willingness of patients to self‐sample. Long‐term feasibility in long‐time diabetes patients warrants particular attention, including wound healing, scarring from repeated sampling^17^, ^51^, infection risk, and collection success in individuals with polyneuropathy or microcirculatory dysfunction. Second, cost‐effectiveness compared to conventional practice‐based venous sampling needs to be investigated, taking into account device costs (currently USD 15–25), staff time, postal processing/shipment costs, and potential savings from reduced practice visits and improved monitoring adherence and their impact on clinical outcomes^52^. Third, the integration into clinical and laboratory workflow requires adaptations or upgrades of laboratory systems, for example, to process smaller sample tubes.

Strengths include the three‐way comparison (mailed capillary, mailed venous, and courier‐transported venous samples), which isolated sampling method effects from postal transport effects, and evaluation under standard postal conditions rather than specialized courier services. In contrast to many previous studies where sampling was performed by healthcare personnel, our study evaluated participant‐performed self‐sampling, and participants subsequently mailed their samples via standard postal services.

However, some limitations of the study should be considered. Insufficient blood volume prevented us from completing metabolic analyses in 22% of samples, which reflects both challenges of SBC in practice. Future developments should aim to optimize the self‐collection process to minimize the rate of insufficient samples. Patient acceptance, preference, and ability to perform self‐sampling, especially among individuals with diabetes complications (e.g., polyneuropathy), and long‐term feasibility were not assessed in this study. Furthermore, we have only controlled for the length of transport but not for other factors that might have impacted sample quality including vibration and climate. As the study was conducted between February and June, seasonal and weather‐related influences on sample quality cannot be excluded. Finally, the generalizability of our findings may be limited by the selection of study participants. The study was conducted in a single region with two general practices; generalizability to other healthcare systems, postal services, and climatic conditions may require further validation. While we included participants with a broad spectrum of HbA1c and creatinine values typical for a general practice setting, the study population may not be representative of all patients.

## CONCLUSIONS

Our study demonstrates high concordance of HbA1c and creatinine values between SBC samples sent by mail and venous blood samples using the Tasso+ device. Lipid parameters also showed good concordance, though with slightly greater variability. Self‐collected capillary blood samples sent by standard mail can be a reliable method for monitoring these parameters in diabetes management. By eliminating practice visits for blood draws, this approach could reduce barriers to diabetes monitoring for patients with transportation difficulties, inflexible work schedules, or living distant from healthcare facilities. Mailed self‐sampling could supplement virtual diabetes check‐ins, enabling remote care for stable patients between in‐person visits. While our findings support the analytical reliability of this method, further evaluation of patient acceptance, cost‐effectiveness, and impact on clinical outcomes such as glycemic control should be conducted prior to implementation into routine care.

## DISCLOSURE

The authors declare no conflict of interest.

Approval of the research protocol: N/A.

Informed consent: Informed consent was obtained from all individual participants included in the study.

Approval of the research protocol: N/A.

Animal studies: N/A.

## FUNDING

The Blood‐it‐yourself study as part of the Blut‐Mobil project was funded through the European Social Funds (ESF), Grant No.: ZAM 5‐87006761. The study funder was not involved in the design of the study; the collection, analysis, and interpretation of data; writing the report; and did not impose any restrictions regarding the publication of the report.

## Data Availability

The datasets generated and/or analyzed during the current study are available from the corresponding author on reasonable request.
